# Retrospective analysis of vertical Hepatitis C exposure and infection in children in Western New York

**DOI:** 10.1186/s12876-023-02871-8

**Published:** 2023-07-17

**Authors:** Ndeye Licka Dieye, Mine Varol, Shauna C. Zorich, Amy E. Millen, Karl O. A. Yu, Oscar G. Gómez-Duarte

**Affiliations:** 1 International Enteric Vaccine Research Program (IEVRP), Division of Pediatric Infectious Diseases, The State University of New York (SUNY) at Buffalo, Buffalo, NY USA; 2 Department of Epidemiology and Environmental Health, School of Public Health and Health Professions, State University of New York (SUNY) at Buffalo, Buffalo, NY USA

**Keywords:** Hepatitis C, Exposure, Infection, Infant, Western New York, Screening, Vertical transmission

## Abstract

**Background:**

Vertical transmission of hepatitis C virus (HCV) is the primary cause of hepatitis C in the pediatric population. Nonetheless, only a small proportion of HCV-exposed children are tested. This study aimed to measure the proportion of HCV-exposed children tested and infected in Western New York and to identify factors influencing the odds of testing and infection in this population.

**Methods:**

This was a 11-year retrospective chart review study in which clinical, demographic, and behavioral data for HCV-exposed children and their mothers were collected. This period included year 2019 when a hepatitis C program began promoting early hepatitis C screening among infants born to mothers positive for hepatitis C. PCR-based detection of hepatitis C was used for children under 18 months of age and antibody testing for children above 18 months of age, followed by PCR if the antibody testing was positive. Logistic regression models were used to determine which characteristics associate with testing and infection status.

**Results:**

From a total of 133 children evaluated in clinic for hepatitis C from 2011 to 2021, 96.2% (128/133) were seen from 2019 to 2021. Among the 133 HCV-exposed children in our sample, 72.1% (96/133) were tested for HCV, 62.4% (83/133) were tested by PCR, 9.0% (12/133) tested by antibody, and 5.2% (5/95) of those tested were infected. Only one child out of 12 was positive for hepatitis C antibody yet, subsequent PCR testing was negative in this child. Among all five hepatitis C infected children, four were diagnosed with neonatal abstinence syndrome, five had maternal history of illicit drug use, one had maternal history of HIV infection, and all of them were identified after the hepatitis C program open in 2019. The odds of a child being tested were lower for those accompanied by their biological mother at their clinic visit (odds ratio, 0.16; 95% CI, 0.06–0.45).

**Conclusions:**

Screening programs on hepatitis C vertical transmission improved detection of hepatitis C among exposed children. The proportion of children born to mothers with hepatitis C in Western New York that were positive for hepatitis C was 5.2%, suggesting that similar proportion of exposed infants born before 2019 were lost for follow up.

## Background

Hepatitis C infection is a leading cause of liver diseases such as cirrhosis and hepatocellular carcinoma. It is a significant cause of morbidity and mortality worldwide, with approximately 71 million people infected, 3.26 million of them being children [1, 2]. Hepatitis C virus (HCV) can be transmitted vertically from mother to child during the pregnancy or birth [3, 4]. Viral transmission was not influenced by mother’s age, mode of delivery, genotype or type of feeding [5]. Perinatal risk factors associated with vertical transmission included duration of membrane rupture and internal fetal monitoring [6]. According to a meta-analysis, vertical transmission is estimated to occur in 5.8% (95% confidence interval [CI], 4.2–7.8%) of children born to women who are infected with HCV, but the risk reaches 10.8% (95% CI, 7.6–15.2%) when the mother is coinfected with HIV [7]. Despite an increased understanding of the rate of hepatis C vertical transmission and epidemiological risk factors associated with transmission, there is limited information on the mechanisms and timing of transmission. It is also unclear which proportion of vertical transmission happens prior or during labor and delivery.

Screening of hepatitis C-exposed infants in the US follows the American Academy of Pediatrics guidelines recommending HCV antibody testing after 18 months of age [8, 9]. Testing rates for children at risk of vertical transmission in the United States vary between 8% and 45%, likely because there is no universally accepted testing or screening protocol and as a result many of these children are lost for follow up [10]. Alternative screening protocols, including HCV PCR testing before 18 months have been proposed as a way to have early diagnosis of perinatally acquired infection and improve HCV surveillance given the substantial loss to follow-up at ≥ 18 months of age [11]. In New York State, studies on the burden of hepatitis C among HCV-exposed children are limited to hospitals in New York City [12, 13]. In 2019 a hepatitis C screening program was established in Western New York to evaluate hepatitis C-perinatally exposed children and to follow those infected for subsequent treatment. Infants less than 18 months of age were tested for hepatitis C by nucleic acid testing and children older than 18 months of age were screened using antibody testing. The program encouraged practicing obstetricians, neonatologists, and pediatric primary care providers in Western New York (WNY) county hospitals to refer hepatitis C exposed infants to the pediatric hepatitis C clinic, a clinic associated with the university pediatric specialty clinics and university children’s hospital in Buffalo, NY. Referral was facilitated by the introduction of hepatitis C testing of pregnant women during prenatal screening in NY.

This study aimed to estimate the proportion of vertically acquired HCV infections during the past ten years among HCV-exposed children residing in Western New York and to identify demographic, clinical, or behavioral characteristics of mothers or children that may be associated with HCV testing and infection. This study also compared the proportion of infants screened before and after the implementation of a program on HCV-exposed children in Buffalo, NY in 2019.

## Methods

### Study population

This is a retrospective chart review study to evaluate the proportion of vertical hepatitis C exposure and infection in infants and children in Western New York. The study population comprised children born to women with positive HCV serology tests who resided in Western New York and who visited pediatric specialty clinics in Buffalo, NY for medical care between January 2011 and December 2021. A hepatitis C program initiated in 2019 opened a pediatric hepatitis C clinic that invited local hospitals and primary care providers to refer hepatitis C-perinatally exposed infants for screening. This clinic was part of the pediatric specialty clinics and university children’s hospital of Buffalo, NY. Information collected from children aged 5 years or younger at their initial visit to the clinic was included in the analyses. Comparing data before and after implementation of the hepatitis C program permitted obtaining information on the gap in hepatitis C screening among HCV-exposed infants in Western New York. The study and protocols were approved by the Institutional Review Board at the University at Buffalo (IRB ID number MOD00010301). A HIPAA waiver was used to access information from electronic medical records. To ensure the confidentiality of collected data, all information was stored at secure locations and all database were password protected. Access to databases was limited to approved investigators only.

Inclusion criteria for enrolment were: (i) Infants evaluated at the pediatric specialty clinics, including the pediatric hepatitis C clinic, between January 2011 and December 2021, (ii) Infants and children less than 5 years of age at the time of first clinic visit during that 10-years period; (iii) i) Residence within the New York State, iv) Infants and children perinatally exposed to HCV from HCV serology positive mothers, and v) Infants born to mothers with or without HIV infection. Exclusion criteria were: (i) Child’s age above 5 years old; (ii) Infants and children residing outside New York State; (iii) Infants and children not being perinatally exposed to or infected with HCV.

### Approach to defining Hepatitis C-exposed and Hepatitis C-infected children

A child with vertical hepatitis C exposure was defined as a child born to a woman with a positive HCV antibody test result or a woman with a previous diagnosis of HCV in her medical record. Medical charts from HCV-exposed children were identified on the basis of diagnosis of hepatitis C-perinatal exposure, maternal HCV status noted in their medical record, or the International Classification of Diseases 9th revision (ICD-9) or ICD-10 codes for hepatitis-perinatal exposure. ICD-10 code Z20.5 was used for vertical hepatitis C exposure and ICD-9 code 070.70 for unspecified hepatitis C. Medical charts with diagnosis that included the word hepatitis were also evaluated. Medical charts that fit the definition of hepatitis C-exposed infants/children were selected for further analysis.

Hepatitis C exposed infants and children seen at the pediatric specialty clinics, including the pediatric hepatitis C clinic opened in 2019, had a referral from the hospital where they were born or a referral from the primary care provider. At clinic, all patients underwent a clinical evaluation with physical exam and at the end of the visit parents or guardians received a laboratory requisition for hepatitis C testing, and a request for a follow up visit. Infants born premature and admitted to the neonatal intensive care unit at the university pediatric hospital in Buffalo, NY had testing done in-house once they reached 2 months of age. The hepatitis C testing included HCV antibody testing for children 18 months of age. If the antibody test result was nonreactive for hepatitis C, the patients were considered clear of infection and no further follow up was recommended. For infants less than 18 months of age, two HCV PCR tests were recommended, one test to be done at 2 months and the second test done 2 months later. Children were considered negative for hepatitis C if one PCR tests was negative for hepatitis C. Children above 18 months of age were diagnosed with hepatitis C if the antibody test was positive and a subsequent HCV PCR was also positive. Infants less than 18 months of age were considered infected with hepatitis C if the first HCV PCR was positive and a second confirmatory HCV PCR was also positive. All patients with confirmed diagnosis of hepatitis C had parents or guardians informed about the disease and were invited to continue to attend the clinic for clinical evaluations, treatment and counselling. The HCV antibody and HCV PCR testing systems available to patients were commercially approved by the New York State and were available at local laboratories within the WNY area. The HCV antibody test was a laboratory-based assay performed at the Kaleida Health Center for Laboratory Medicine (Williamsville, NY). The HCV antibody testing result was reported as: (i) nonreactive, if the signal to cut-off ratio (S/CO ratio) was 0.0 to 0.79; (ii) equivocal, if S/CO ratio was 0.80 to 0.99; and (iii) reactive, if S/CO ratio was > 1.0. The HCV PCR testing used on most patient blood specimens was the Cobas ® TaqMan ® HCV test, v2.0 performed at the Kaleida Health Center for Laboratory Medicine (Williamsville, NY). The quantitative range of the assay was 15 units/mL to 100 million units/mL, and any testing below the level of detection was considered HCV negative.

### Data extraction

Data extraction included screening of pediatric charts that contained unique ICD-10 codes for hepatitis or hepatitis C and/or keywords in the discharge diagnosis including hepatitis, hepatitis C, hepatitis exposure. Those pediatric charts on patients that fulfill inclusion and exclusion criteria were evaluated individually to retrieve pediatric study data. We also attempted to obtain biological maternal data by searching the EMRs of those mothers who delivered the infant at the local children’s university hospital of Buffalo. Biological mother information on infants born at hospitals with EMRs not available to investigators was not retrieved. EMR data from infants and biological mothers extracted and collected in a database included demographic, clinical, testing results, epidemiological risk factors and behavioral variables. Data on biological mothers were collected from the mother’s EMR if this data was missing in the child’s EMR. The only information collected from non-biological mother, including foster parents, guardians, and adaptive parents, was whether these non-biological parents accompanied that child to clinic. The clinical and demographic characteristics collected from hepatitis C-exposed infants and children included: age at the first visit to the pediatric specialty clinics, sex, race, ethnicity, route of birth delivery, history of neonatal disorders requiring hospitalization at birth (including respiratory distress and neonatal abstinence syndrome), prematurity (i.e., birth before 37 weeks of gestation), born small for gestational age (birth weight below the 10th percentile), who accompanied them to the clinic, and HCV test results. The characteristics of the mothers that were obtained for further analysis included: age at labor, race, ethnicity, level of education, whether they received prenatal care, whether they had any psychiatric diagnoses, tobacco use, tetrahydrocannabinol (THC) use, recreational drugs use, and/or injection drugs or had undergone opioid agonist therapy, human immunodeficiency virus (HIV) status, whether they have had multiple sex partners or a partner with HCV infection, whether they had undergone surgery (e.g., cesarean), needlestick/mucosal exposure (including for tattoos), whether they have ever been incarcerated, whether they ever had a blood transfusion, and their HCV infection and treatment status.

### Statistical analysis

Data obtained from electronic medical records was kept in excel databases. Data were analyzed using descriptive statistics, and regression analysis using RStudio version 2023.6.0.421 (RStudio Team 2023). Central tendency and dispersion values were calculated for all variables analyzed. Counts and percentages were determined for the categorical variables. Differences in variables between groups of children tested or not for HCV and between those infected or not with HCV were compared using chi-square or Fisher exact where appropriate. The significance level was set at 0.05. Variables associated with testing or infection status at a *P* value of ≤ 0.05 were included in logistic regression models to estimate the crude odds ratios and 95% CIs for infant/child being tested for HCV and being positive for HCV.

Single hepatitis C PCR test sensitivity and specificity was calculated using as a gold standard two hepatitis C PCR consecutive tests (two negative PCR results to rule out hepatitis C infection or two positive PCR results to confirm hepatitis C). Based on the sensitivity and the specificity values, we used the results of the first test to define the infection status of children who were tested using PCR assay.

## Results

### Study cohort

**Fig. 1:**
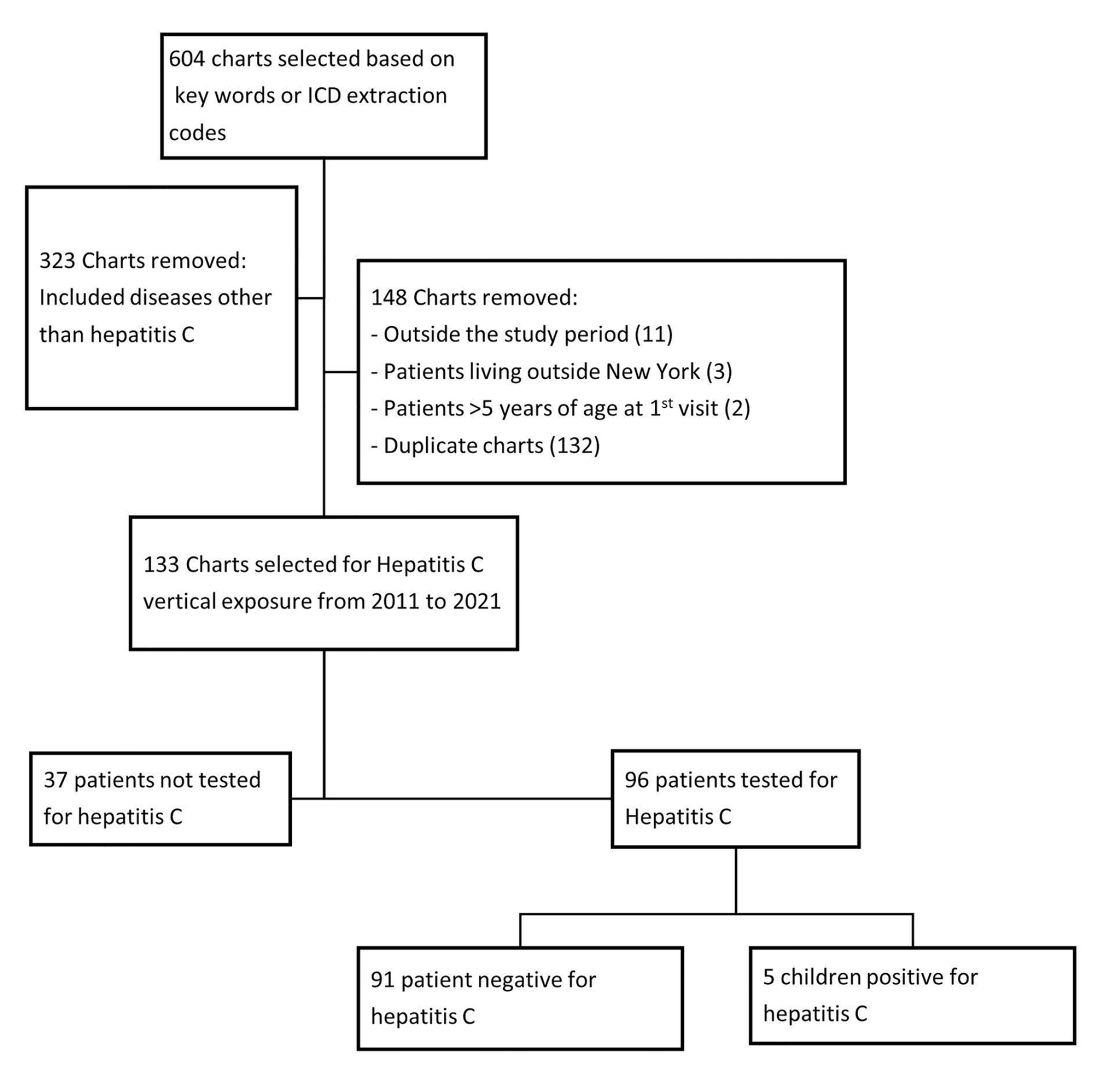
Retrospective study flowchart to evaluate vertical hepatitis C exposure and infection among children in Western New York. Medical charts were reviewed retrospectively from years 2011 to 2021. ICD, International Classification of Diseases; MRN, medical record numbers

Of the 604 medical records identified during the study period, 471 medical record numbers were excluded because they either did not meet the inclusion criteria or were duplicates. A final sample of 133 children with vertical exposure to HCV was obtained. Of these, 96 had been screened for HCV using an antibody test or at least one PCR; 5 (5.2%) tested positive for HCV (Fig. 1). The characteristics of the overall population of children are presented in Table 1. The predominant racial and ethnic categories among hepatitis C-exposed infants and children in this study were 74% White, 13.5% Black, 3% American Indian/Alaska Native and 6.8% Hispanic. Among biological mothers of these children, the predominant racial and ethnic categories were 54% White, 6.3% Black, 2.3% American Indian/Alaska Native and 6% Hispanic.

**Table 1:** Demographic and clinical characteristics of HCV-exposed children (*n* = 133)

Characteristic	*n* (%)
Sex	
Male	77 (57.9)
Female	56 (42.1)
Age at first visit (years)	
< 1	119 (89.5)
1 to < 3	8 (6.0)
3 to 5	5 (4.5)
Race	
American Indian or Alaska Native	3 (2.3)
Asian	3 (2.3)
Black or African American	18 (13.5)
White	99 (74.4)
Unknown	10 (7.5)
Ethnicity	
Hispanic/Latino/Latina	9 (6.8)
Non-Hispanic/Latino/Latina	119 (89.5)
Unknown	5 (3.8)
Route of delivery	
Vaginal	65 (48.9)
Cesarean	49 (36.8)
Unknown	19 (14.3)
Neonatal abstinence syndrome	
Yes	64 (48.1)
No	53 (39.8)
Unknown	16 (12)
Small for gestational age	
Yes	27 (20.3)
No	53 (39.8)
Unknown	53 (39.8)
Prematurity	
Yes	26 (19.5)
No	57 (42.9)
Unknown	50 (37.6)
Accompanying person	
Biological mother	82 (61.7)
Foster parent	31 (23.3)
Guardian (relative or state)	13 (9.8)
Other	4 (3.0)
Unknown	3 (2.3)

**Fig. 2:**
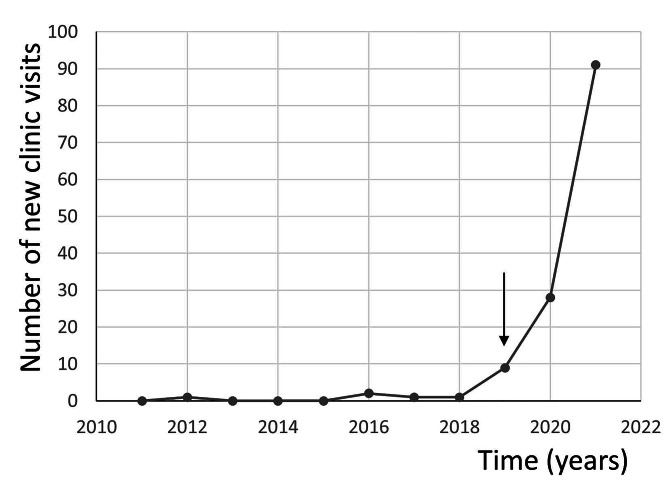
Distribution of new annual visits for hepatitis C-perinatal exposure in children less than 5 years of age. Children were seen by the pediatric infectious diseases clinic providers in Buffalo, NY from 2011 to 2021. The red arrow indicate the time when a hepatitis C-perinatal exposure program was initiated at the pediatric infectious diseases clinic

Five (3.8%) children with hepatitis C-perinatal exposure visited the pediatric specialty clinics from 2011 to 2018 and the remaining 128 (96.2%) children visited these clinics from 2019 to 2021 (Fig. 2). All 5 hepatitis C positive children were identified from 2019 to 2021. Among 12 hepatitis C-exposed children 18 months of age and older, 11 (91.7%) tested negative for hepatitis C antibody test and only one (8.3%) was tested positive yet, this child tested negative for HCV by PCR testing.

### HCV-testing in exposed children

We compared the characteristics of mothers of children that were tested for HCV and those that were not (Table 2). Greater proportions of children who were tested were accompanied to the clinic by someone other than their biological mother (i.e., foster parent, guardian, or other) (*P* < .001) (Table 3). According to the logistic regression models, the odds of a child being tested were significantly lower for those accompanied to the clinic by their biological mother (odds ratio, 0.16; 95% CI, 0.06–0.45) (Table 4).

**Table 2:** Testing status of HCV-exposed children according to their mothers’ characteristics

Characteristic	*n* (%) for each characteristic		*P* value^a^
	Not tested (*n* = 37)	Tested (*n* = 96)	
Age at labor (yr)			0.177
15–24	0	10 (10.4)	
25–34	19 (51.4)	55 (57.3)	
35–44	3 (8.1)	10 (10.4)	
Unknown	15 (40.5)	21 (21.9)	
Race			0.486
American Indian or Alaska Native	0	2 (2.1)	
Asian	0	4 (4.2)	
Black or African American	1 (2.7)	6 (6.3)	
White	24 (64.9)	52 (54.2)	
Unknown	12 (32.4)	32 (33.3)	
Ethnicity			0.720
Hispanic/Latino/Latina	3 (8.1)	6 (6.3)	
Non-Hispanic/Latino/Latina	25 (67.6)	61 (63.5)	
Unknown	9 (24.3)	29 (30.2)	
Prenatal follow-up			. 498
Yes	17 (45.9)	47 (49.0)	
No	10 (27.0)	20 (20.8)	
Unknown	10 (27.0)	29 (30.2)	
Ever used tobacco/THC			. 506
Yes	21 (56.8)	54 (56.3)	
No	2 (5.4)	12 (12.5)	
Unknown	14 (37.8)	30 (31.3)	
Ever used illicit drug			0.572
Yes	15 (40.5)	50 (52.1)	
No	4 (10.8)	23 (24.0)	
Unknown	18 (48.6)	23 (24.0)	
Ever used injection drug			0.118
Yes	18 (48.6)	40 (41.6)	
No	4 (10.8)	24 (25.0)	
Unknown	15 (40.5)	32 (33.3)	
Received opioid agonist therapy			. 087
Yes	21 (56.8)	39 (40.6)	
No	5 (13.5)	25 (26.0)	
Unknown	11 (29.7)	32 (33.3)	
HIV status			0.660
Infected	2 (5.4)	4 (4.2)	
Not infected	28 (75.7)	77 (80.2)	
Unknown	7 (18.9)	15 (15.6)	
HCV viremic status			0.737
Viremic	13 (35.1)	36 (37.5)	
Not viremic	18 (48.6)	40 (4.6)	
Unknown	6 (16.2)	20 (20.8)	
HCV treatment status			0.977
Treated	8 (21.6)	20 (20.8)	
Not treated	13 (35.1)	33 (34.4)	
Unknown	16 (43.2)	43 (44.8)	

^a^According to chi-square and Fisher tests

^*^P value ≤ 0.05

**Table 3:** Testing status of HCV-exposed children according to their clinical characteristics

Characteristic	*n* (%)		*P* value ^a^
	Not tested (*n* = 37)	Tested (*n* = 96)	
Neonatal abstinence syndrome			0.270
Yes	23 (62.2)	41 (42.7)	
No	14 (37.8)	39 (40/6)	
Unknown	0	16 (16.7)	
Small for gestational age			
Yes	12 (32.4)	15 (15.6)	0.276
No	17 (45.9)	36 (37.5)	
Unknown	8 (21.6)	45 (46.9)	
Prematurity			0.013
Yes	4 (10.8)	22 (22.9)	
No	25 (67.6)	32 (33.3)	
Unknown	8 (21.6)	42 (43.8)	
Accompanying person			< 0.001^*^
Biological mother	32 (86.5)	50 (52.1)	
Foster parent	1 (2.7)	30 (31.3)	
uardian (relative or state)	2 (5.4)	11 (11.5)	
Other	1 (2.7)	3 (3.1)	
Unknown	1 (2.7)	2 (2.1)	

^a^According to chi-square, Fisher, and *t* tests

^*^ P value ≤ 0.05

**Table 4:** Odds of being tested for HCV according to logistic regression model

Category	
	Crude
Accompanying person	
Biological mother	0.14 (0.04–0.40)^a^
Foster parent, guardian, or other	Reference

^a^Statistically significant

### HCV-infection in tested children

Specificity and sensitivity of a single PCR assay for detecting HCV infection were both 100% (Table 5). Similarly, the positive predictive value and the negative predictive value were 100%. Among epidemiological risk factors for vertical transmission, we found the risk of hepatitis C vertical transmission was not higher among children born to an HIV-infected mother (*P* = .229) or born to a mother with positive viremic status (*P* = .101) (Table 6). No increased risk of hepatitis C vertical transmission was identified when route of delivery (P = .343), prematurity (P = > 0.999) or gestational age (> 0.999) was evaluated (Table 7) compared to children who tested negative.

**Table 5:** Hepatitis C PCR test sensitivity and specificity.^a^

Test result^b^	Infected	Not infected
Positive	5	0
Negative	0	34
**Statistic**	**Value**	
Sensitivity	100%	
Specificity	100%	
Positive predictive value	100%	
Negative predictive value	100%	

^a^The sensitivity and specificity of a single PCR testing, compared to gold standard, defined as two consecutive PCR tests, to detect HCV were calculated as follows: sensitivity = true positive/(true positive + false negative); specificity = true negative/(true negative + false positive). ^b^PCR negative or positive results were based on the quantitative assay ranging from 15 units/mL to 100 million units/mL. Any testing below the level of detection was considered HCV PCR negative

**Table 6:** Infection status of HCV-tested children according to their mothers’ clinical characteristics

Characteristic	*n* (%) for each characteristic		*P* value^a^
	HCV negative (*n* = 91)	HCV positive (*n* = 5)	
HIV status			0.229
Infected	3 (3.3)	1 (20.0)	
Not infected	73 (80.2)	4 (80.0)	
Unknown	15 (16.5)		
HCV viremic status			0.101
Viremic	33 (36.3)	0	
Not viremic	40 (43.9)	3 (60.0)	
Unknown	18 (19.8)	2 (40.0)	
HCV treatment status			. 627
Treated	18 (19.8)	2 (40.0)	
Not treated	31 (34.1)	2 (40.0)	
Unknown	42 (46.1)	1 (20.0)	

^a^According to chi-square and Fisher tests

^*^ P value ≤ 0.05

**Table 7:** Infection status of HCV-tested children according to their clinical characteristics

Characteristic	*n* (%)		*P* value^a^
	HCV negative (*n* = 91)	HCV positive (*n* = 5)	
Route of delivery			
Vaginal	50 (54.9)	2 (40.0)	0.343
Cesarean	26 (28.6)	3 (60.0)	
Unknown	15 (16.5)	0	
Prematurity			> 0.999
Yes	21 (23.1)	1 (20.0)	
No	30 (33.0)	2 (40.0)	
Unknown	40 (43.9)	2 (40.0)	
Small for gestational age			> 0.999
Yes	14 (15.4)	1 (20.0)	
No	34 (37.4)	2 (40.0)	
Unknown	43 (47.2)	2 (40.0)	

^a^According to chi-square and Fisher tests

^*^P value ≤ 0.05

Overall, 5 (5.2%) infants/children out of 96 were positive for hepatitis C by PCR, 3 of them were female, 3 of them were diagnosed with neonatal abstinence syndrome, and only one was born to mother with HCV/HIV coinfection (Table 8).

**Table 8:** Summary features of Hepatitis C perinatally-exposed and infected children in Western New York

	Subjects				
Features	1	2	3	4	5
Prematurity^b^	No	Yes	No	NK	No
Small for gestational age^c^	Yes	No	NK	NK	No
NAS^d^	Yes	Yes	No	NK	Yes
Maternal history of illicit drug	Yes	Yes	Yes	NK	Yes
Maternal HIV	No	Yes	No	No	No

^a^ Sex abbreviation are M for male and F for female. ^b^ Prematurity defined as gestational age less than 37 weeks gestation. ^c^Small for gestational age refers to weight at birth below the 10th percentile. ^d^NAS refers to neonatal abstinence syndrome

## Discussion

Limited information is available about the number of hepatitis C-exposed and -infected infants and children in the US as many of them are lost for follow up. We showed that 5.2% of hepatitis C-exposed infants and children in this study vertically acquired hepatitis C from their mothers. This study also showed that the hepatitis C screening program implemented in 2019 in the city of Buffalo, NY for hepatitis C-exposed infants increased the number of patients screened when comparing the proportion of infants screened before and after 2019. More importantly, it allowed the recognition of children with hepatitis C infection not previously reported from this geographic location to the New York State Department of Health. This study also suggested that a high number of hepatitis C vertically exposed infants born before 2019 were probably lost for follow up. There is evidence that hepatitis C-exposed infants/children prior to 2019 were not screened for hepatitis C. First, no hepatitis C in children was reported to the New York State Health Department from Wester New York before 2019. Second, pediatric infectious diseases and pediatric gastroenterologist specialists in Western New York had a very low number of patients evaluated for hepatitis C according to the search of electronic medical records that investigators in the study had access to. Furthermore, there was no mandatory hepatitis C screening of pregnant women prior to 2019.

A total of 96 (72.1%) hepatitis C-exposed children in our study sample were tested for HCV. This is a higher rate of testing compared to rate reported in previous studies, including the state of Wisconsin and the city of Boston (34% and 45%, respectively) [14, 15]. In this study we considered “tested” those patients who have received at least one PCR or one antibody test. If our definition were based on two PCR tests for children ≥ 2 months old followed by an antibody testing at ≥ 18 months old or an antibody test for children ≥ 18 months old [8], our testing rate would be 37.6% (50/133), which is more in line with the previously reported rates.

The PCR test results revealed that 5.2% of hepatitis C-exposed children become infected, which is consistent with the results of previous studies [16]. When a single HCV PCR testing was compared to a two HCV PCR gold standard testing, we found that the sensitivity and specificity were 100%. Overall, the sensitivity and the specificity of a single HCV PCR test are very high yet two HCV PCR are still recommended to confirm or rule out hepatitis C infection. Although the sensitivity of the PCR test is low at birth and is around 79% at 1 month of age [17], the children tested via PCR in our study were on average older than 2 months when being tested. Negative PCR tests in our patients could reflect clearance of the virus, which occurs spontaneously in 25–50% of children [18].

The odds of a child being tested for HCV were associated with who accompanied them to the clinic and whether their mother was a tobacco user. Specifically, children accompanied by someone other than their biological mother were more likely to be tested. One possible explanation to this finding is that foster parents and guardians are legally required to meet all health requirements of the child. In contrast, a proportion of biological mothers may be limited in their ability to address infant health care needs due to deterrent social determinants of health including, but not limited to, injection drug use disorder, incarceration, limited access to health care, limited transportation, among others.

Maternal HIV/HCV coinfection was not associated with an increased risk of hepatitis C vertical transmission, a finding that was not consistent with studies reporting how HIV/HCV coinfection increases the risk on hepatitis C infection in the offspring [19]. The only child (older than 18 months of age) that tested positive for hepatitis C antibody test and tested negative for HCV PCR was born to a mother with HCV/HIV coinfection. Inclusion of this child in the group of hepatitis C positive children would have resulted in an association of maternal HIV/HCV coinfection with and increased risk of hepatitis C vertical transmission in this study. The occurrence of HCV infection in children was independent of the mother’s HCV RNA positive status (HCV RNA positive by PCR) and the mother’s HCV RNV viral load was also not associated with higher risk of vertical transmission as previously reported [20]. In contrast, HCV vertical transmission was not associated with delivery route which is consistent with prior reported studies evaluating HCV vertical transmission, one on 214 mother-child pairs and the other, a meta-analysis, evaluating a total of 614 mother-child pairs, both of which consistently demonstrated that C-section did not decrease the perinatal HCV transmission from HCV RNA + HIV- mothers to infants [21, 22].

Most children in this study had a clinic visit for hepatitis C after 2019, the same year the hepatitis C-perinatal exposure program started and local hospital providers were encouraged to refer hepatitis C-perinatally exposed infants to clinic for screening. The number of children exposed to or infected by HCV that remain unaccounted for in the US is expected to be high. Based on information we provided, an extremely low number of HCV-exposed infants in WNY were screened for hepatitis C prior to 2019 compared to the infants screened after implementation of the hepatitis C program. The main reasons for these findings were that hepatitis C was not routinely done on pregnant women in the state of NY, the American Academy of Pediatric did not recommended early hepatitis C screening among HCV-exposed infants and the recommended screening with hepatitis C serology after 18 months of age resulted in HCV-exposed infants being lost for follow up. Study data strongly suggests that unless hepatitis C screening programs are implemented in the US, a large proportion of HCV-exposed infant will be left unscreened, undiagnosed, and even worse untreated. We believe that hepatitis C programs targeting exposed or infected infants are essential for hepatitis C detection, diagnosis, treatment, and eventual elimination of this bloodborne infection in the US [23]. The Center for Disease Control and Prevention has drafted a recommendation on hepatitis C-exposed infant for early screening as early as 2 months of age by HCV PCR testing [24].

This study had some limitations. The study had a small sample size which may have limited the likelihood of finding otherwise statistically significant associations with HCV vertical transmission, including HCV/HIV coinfection or high HCV viral load in the biological mother. There was missing information in the hepatitis C-exposed infants/children EMR as well as their biological mother’s EMRs, making impossible to acquired complete data for the variables of interest. Maternal medical records were not available for many of the children, particularly for those born at hospitals with electronic medical records not available to investigators of this study.

Regarding missing hepatitis C testing, we recorded 37 infants/children that did not performed hepatitis C testing after evaluation for hepatitis C in clinic. One possible explanation for this outcome was the unavailable blood draw capabilities in clinic and the need to send patient to an outside laboratory facility. This blood draw clinic limitation may have preferentially affected testing of infants/children accompanied by their biological mothers since finding a laboratory away from clinic may have added difficulties to a parent already overwhelmed with negative social determinants of health.

### Conclusions and future work

To our knowledge, this is the first study focused on vertical hepatitis C transmission and the association between testing/infection status and children’s and mother’s characteristics in Western New York. Most of the hepatitis C-exposed children were identified after a hepatitis C pediatric clinic program was opened in Western New York in 2019 and, among them, 5.2% were infected with hepatitis C. The results may raise awareness about HCV vertical transmission and the importance to conduct early follow up of infants born to HCV-infected women. Prior to the implementation of the screening program, few children were screened for hepatitis C in WNY based on evaluation of EMR of our pediatric specialty clinics. Before 2019, the American Academy of Pediatrics recommendation was to screen hepatitis C-exposed children after 18 months of age, and those children would be referred to our pediatric specialty clinics for testing. From 2011 to 2019, only 5 out of 133 children were screened compared to 128 out of 133 after 2019 to 2021.

Expanding epidemiological surveillance program on hepatitis C among infants, children, adolescents, pregnant women, and people at risk are essential to better address the needs of the community. We believe that hepatitis C surveillance programs are needed in all states in the US and worldwide. These programs will considerably contribute to achieving the CDC’s and the World Health Organization’s goal of eradicating hepatitis C.

## Data Availability

The dataset supporting the conclusions of this study is included within the article. Additional data is available upon request by contacting the corresponding author Oscar G. Gomez at oscargom@buffalo.edu, as stated in the protocol approved by the State University of New York (SUNY) University at Buffalo Institutional Review Board.

## Abbreviations

CDC: Center for Disease Control and Prevention
CI: Confidence interval
HCV: Hepatitis C virus
HIPAA: Health Insurance Portability and Accountability Act of 1996
HIV: Human immunodeficiency virus
ICD: International Classification of Diseases
ICD-9: International Classification of Diseases 9th revision
ICD-10: International Classification of Diseases 10th revision
IRB: Institutional Review Board
THC: Tetrahydrocannabinol
WHO: World Health Organization

## References

[1] Epstein RL, Espinosa C (2021). Hepatitis C Virus in Neonates and Infants. Clin Perinatol.

[2] World Health Organization (2017). Global hepatitis report 2017.

[3] Chigbu DI, Loonawat R, Sehgal M, Patel D, Jain P (2019). Hepatitis C virus infection: Host^–^Virus Interaction and Mechanisms of viral persistence. Cells.

[4] Chen SL, Morgan TR (2006). The natural history of hepatitis C virus (HCV) infection. Int J Med Sci.

[5] Syriopoulou V, Nikolopoulou G, Daikos GL, Theodoridou M, Pavlopoulou I, Nicolaidou P (2005). Mother to child transmission of hepatitis C virus: rate of infection and risk factors. Scand J Infect Dis.

[6] Mast EE, Hwang L-Y, Seto DSY, Nolte FS, Nainan OV, Wurtzel H (2005). Risk factors for perinatal transmission of hepatitis C virus (HCV) and the natural history of HCV infection acquired in infancy. J Infect Dis.

[7] Benova L, Mohamoud YA, Calvert C, Abu-Raddad LJ (2014). Vertical Transmission of Hepatitis C Virus: systematic review and Meta-analysis. Clin Infect Dis.

[8] Kimberlin DW, Brady MT. Recommendations for care of children in special circumstances; hepatitis C. Red Book: 2015 Report of the Committee on Infectious Diseases. 2015.

[9] Mack CL, Gonzalez-Peralta RP, Gupta N, Leung D, Narkewicz MR, Roberts EA (2012). NASPGHAN practice guidelines: diagnosis and management of hepatitis C infection in infants, children, and adolescents. J Pediatr Gastroenterol Nutr.

[10] Bhardwaj AM, Mhanna MJ, Abughali NF (2021). Maternal risk factors associated with inadequate testing and loss to follow-up in infants with perinatal hepatitis C virus exposure. NPM.

[11] Gowda C, Smith S, Crim L, Moyer K, Sánchez PJ, Honegger JR (2020). Nucleic acid testing for diagnosis of perinatally-acquired Hepatitis C virus infection in early infancy. Clin Infect Dis.

[12] Granovsky MO, Minkoff HL, Tess BH, Waters D, Hatzakis A, Devoid DE (1998). Hepatitis C virus infection in the mothers and Infants Cohort Study. Pediatrics.

[13] Kushner T, Park C, Masand D, Wagner B, Grace M, Rosenbluth E (2020). Hepatitis C Seroprevalence among Consecutive Labor and Delivery admissions in two New York City Hospitals. Open Forum Infectious Diseases.

[14] Epstein RL, Sabharwal V, Wachman EM, Saia KA, Vellozzi C, Hariri S (2018). Perinatal transmission of Hepatitis C Virus: defining the Cascade of Care. J Pediatr.

[15] Watts T, Stockman L, Martin J, Guilfoyle S, Vergeront JM (2017). Increased risk for mother-to-infant transmission of Hepatitis C Virus among Medicaid Recipients - Wisconsin, 2011–2015. MMWR Morb Mortal Wkly Rep.

[16] Polywka S, Pembrey L, Tovo P-A, Newell M-L (2006). Accuracy of HCV-RNA PCR tests for diagnosis or exclusion of vertically acquired HCV infection. J Med Virol.

[17] Durmaz O (2012). Hepatitis C infection in childhood. Clin Res Hepatol Gastroenterol.

[18] Indolfi G, Mangone G, Bartolini E, Moriondo M, Azzari C, Resti M (2016). Hepatitis C viraemia after apparent spontaneous clearance in a vertically infected child. Lancet.

[19] England K, Thorne C, Newell M-L (2006). Vertically acquired paediatric coinfection with HIV and hepatitis C virus. Lancet Infect Dis.

[20] Elrazek A, Amer M, El-Hawary B, Salah A, Bhagavathula AS, Alboraie M (2017). Prediction of HCV vertical transmission: what factors should be optimized using data mining computational analysis. Liver Int.

[21] Delotte J, Barjoan EM, Berrébi A, Laffont C, Benos P, Pradier C (2014). Obstetric management does not influence vertical transmission of HCV infection: results of the ALHICE group study. J Matern Fetal Neonatal Med.

[22] Ghamar Chehreh ME, Tabatabaei SV, Khazanehdari S, Alavian SM (2011). Effect of cesarean section on the risk of perinatal transmission of hepatitis C virus from HCV-RNA+/HIV- mothers: a meta-analysis. Arch Gynecol Obstet.

[23] Varol M, Licka Dieye N, Zang M, Handa D, Zorich C, Millen S (2022). Hepatitis C Virus exposure and infection in the Perinatal Period. Curr Pediatr Rev.

[24] Centers for Disease Control and Prevention. CDC Recommendations for Hepatitis C Testing among perinatally exposed Infants and Children-United States, 2023; request for comment and notice of informational Webinar. Fed Reg. 2022;:71330–2.

